# Natural transformation and cell division delay in competent *Staphylococcus aureus*

**DOI:** 10.1128/spectrum.02807-23

**Published:** 2023-10-13

**Authors:** Fedy Morgene, Célia Rizoug Zeghlache, Shi Yuan Feng, Yolande Hauck, Nicolas Mirouze

**Affiliations:** 1 Université Paris-Saclay, CEA, CNRS, Institute for Integrative Biology of the Cell (I2BC), Gif-sur-Yvette, France; Institut Necker Enfants Malades, Paris, France

**Keywords:** *Staphylococcus aureus*, horizonral gene transfer, genetic competence, natural transformation, cell cycle, division inhibition

## Abstract

Genetic competence for natural transformation, considered one of the three main mechanisms leading to horizontal gene transfer in bacteria, is able to promote evolution, through genomic plasticity, and foster antibiotic resistance and virulence factors spreading. Conserved machinery and actors required to perform natural transformation have been shown to accumulate at different cellular localizations depending on the model organism considered. In this study, we investigate the transformation apparatus composition, localization, and dynamics in the human pathogen *Staphylococcus aureus*. We particularly show that most of the natural transformation actors co-localize in clusters. We also reveal that the localization of natural transformation proteins is dynamic, following the cell cycle. Ultimately, the natural transformation apparatus is preferentially established in the vicinity of the division septum. All these results demonstrate that DNA binding, uptake, and recombination are spatially and temporally coordinated to ensure *S. aureus* natural transformation. Finally, we hypothesize that *S. aureus* competent cells would initiate and then block cell division to ensure the success of natural transformation before the final constriction of the cytokinetic ring.

**IMPORTANCE:**

Natural transformation, considered one of the three main mechanisms leading to horizontal gene transfer in bacteria, is able to promote genomic plasticity and foster antibiotic resistance spreading. Conserved machinery and actors required to perform natural transformation have been shown to accumulate at different cellular localizations depending on the model organism considered. Here, we show in the human pathogen *Staphylococcus aureus* that DNA binding, uptake, and recombination are spatially and temporally coordinated to ensure *S. aureus* natural transformation. We also reveal that localization of natural transformation proteins occurs in the vicinity of the division septum allowing *S. aureus* competent cells to block cell division to ensure the success of natural transformation before the final constriction of the cytokinetic ring.

## INTRODUCTION

Natural transformation, alongside conjugation and transduction, is one of the three main mechanisms allowing horizontal gene transfer (HGT) in prokaryotes. Natural transformation refers to the ability displayed by certain bacteria to bind and take up exogenous DNA present in the environment for intracellular recombination with their own chromosome. This mode of HGT is clinically relevant as it is used by important human pathogens to acquire drug resistance or virulence genes (1, 2). Interestingly, while conjugation and transduction rely on mobile genetic elements “attacking” the recipient cell, natural transformation is entirely controlled by a physiological adaptation, called genetic competence, developing in the acceptor bacterium (3).

Many bacterial species are able to enter the competence state to perform natural transformation, one of the latest being the human pathogen *Staphylococcus aureus* (4 – 6). Under laboratory planktonic conditions, competence can be induced in the late exponential phase in nearly 50% of *S. aureus* cells (7). In addition, we have recently shown that competence is induced in response to a decrease in oxygen concentration, an environmental stimulus that *S. aureus* often encounters during the course of an infection (7). Although we have gained important insights into the competence regulatory network at play in *S. aureus*, very little is known about the steps and actors defining natural transformation in this human pathogen (4, 7).

Importantly, the organization of the machinery accomplishing natural transformation steps has been characterized by a number of model organisms (8). First, different structures have been shown to be involved in the initial exogenous DNA binding in various bacterial species. Indeed, while a competence-specific pilus, a structure evolutionary close to type-IV pili and type-II secretion systems, might capture double-strand DNA (dsDNA) from the environment in *Streptococcus pneumoniae* (9), wall teichoic acids produced and modified during competence might represent the initial DNA binding site in *Bacillus subtilis* (10). Uptake of dsDNA then occurs through binding to the membrane-anchored ComEA protein (11) but also requires the competence-specific pilus (12), that might channel DNA through the cell wall to ComEA. The *comG* operon encodes most of the proteins required for the construction of this type IV transformation pilus (13). Assembly of the major pilin ComGC, and potentially additional minor pilins, all matured by the ComC peptidase (14), into this pilus requires the traffic ATPase ComGA (15). Once transported across the cell wall, exogenous dsDNA is converted to single-stranded DNA (ssDNA) (16), which is then taken up into the cytosol through the aqueous transport channel formed by the ComEC integral membrane protein (17, 18). ComFA, a cytosolic ATPase containing helicase-like domains, is thought to facilitate ssDNA internalization (19), through ComEC. Within the cell, the imported ssDNA is protected by a ssDNA-binding protein, SsbB, and the processing protein A, DprA (20) which finally ensures the loading and polymerization of RecA (21), to form presynaptic filaments leading to chromosomal recombinants.

Interestingly, localization of the transformation apparatus has shown that all the steps and actors involved are spatially and temporally closely organized (22, 23). Indeed, exogenous dsDNA naturally binds in the direct vicinity of the proteins involved in its transport (10, 22, 23). In addition, the proteins involved in DNA transport and recombination have also been shown to co-localize (22, 24). Importantly, all these steps and actors have been shown to be organized at different cellular locations in various model organisms: midcell in *S. pneumoniae* (24) and at the cell pole in *B. subtilis* (22). Finally, despite those differences, the establishment of the transformation apparatus has often been associated with the cell cycle and the inhibition of cell division (25, 26).

In this study, we investigated the transformation apparatus composition, localization, and dynamics in *S. aureus*. In particular, we show how ComGA localization is dynamic in space and time, evolving from diffuse in the cytosol to associated with the inner face of the membrane, to finally concentrate in foci next to the division septum. In addition, ComGA localization is coordinated with the cell cycle as most ComGA-expressing competent cells present a forming or completed septum, at the opposite of most non-competent cells. Importantly, ComGA localization evolves with the cell cycle, ultimately accumulating into foci in the direct vicinity of the septum. We finally confirm that representatives of each natural transformation step co-localize with ComGA. On the whole, we propose that the dynamic colocalization of natural transformation machinery next to the septum has multiple roles (i) to spatially coordinate DNA binding, uptake, and processing, (ii) to synchronize these steps with the *S. aureus* cell cycle, and (iii) to impose a delay in cell division, probably through the inhibition of septum synthesis and/or constriction.

## RESULTS

### ComGA exhibits several localization patterns in competent *S. aureus* cells

The traffic ATPase ComGA has been shown to be essential for both DNA binding and transport across the cell wall in *B. subtilis* (27). In addition, as it co-localizes with most of the natural transformation proteins, it has been used as a reference to visualize the transformation apparatus in numerous microscopy studies (10, 22). Therefore, we first investigated ComGA localization in *S. aureus* competent cells produced using our optimized “dilution” protocol (7). To do so, we constructed a translational fusion between the gene encoding the green fluorescent protein (e*gfp*) to the C-terminus of *comGA*, expressed from the natural *comG* promoter (St113, pRIT-P*_comG_-comGA-egfp*).

**Fig 1:**
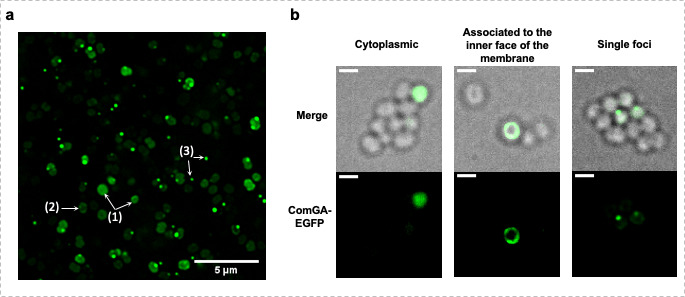
Localization of ComGA-EGFP in competent *S. aureus* cells. (**A**) St113 strain (pRIT-P*_comGA_-comGA-egfp*) was grown to competence in CS2 medium (22 h, 10^−5^ dilution). ComGA-EGFP displays several cellular localizations in *S. aureus* competent cells: cytoplasmic (1), associated with the inner face of the membrane (2), and accumulation in foci (3). Bar = 5 µm (**B**) Examples of the three ComGA-EGFP cellular localizations observed in St113 cultures (pRIT-P*_comGA_-comGA-egfp*). 360 degrees and 3D rotations of these cellular localizations are presented in Fig. S1b and videos S1 through S3. Bar = 1 µm.

First, as expected, the ComGA-EGFP fluorescent signal was present in 19%–23% of the total cells at the entry in the stationary phase (Fig. S1A). Interestingly, in late exponential growth, ComGA-EGFP exhibited several localization patterns in distinct cells (Fig. 1A). Indeed, while in some cells, ComGA-EGFP would be completely cytosolic, it was also found homogeneously localized or concentrated in individual foci at the inner face of the membrane in other competent cells (Fig. 1B, Fig. S1B and Videos S1 through S3). Interestingly, in *B. subtilis*, ComGA has been shown to form individual or multiple foci associated with the inner face of the membrane (22). In comparison, competent *S. aureus* cells would only form a single focus as cells displaying two or more foci only represented 0.8% of all the competent cells (Table S1). Importantly, we ultimately verified that the expression of *comGA*-egfp from the pRIT plasmid was still dependent on SigH (Fig. S2).

### ComGA localization is dynamic

**Fig 2:**
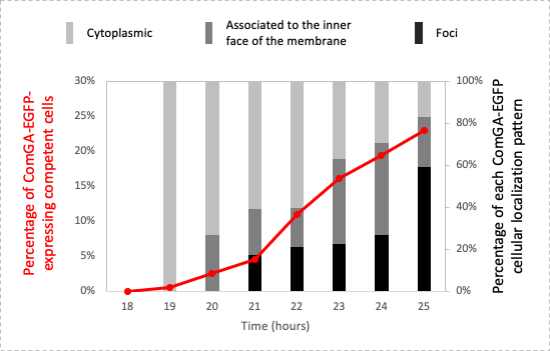
Spatial and temporal localization of ComGA is dynamic in competent *S. aureus* cells. The evolution of ComGA-EGFP localization patterns throughout growth was visualized using the St113 strain (pRIT-P*_comGA_-comGA-egfp*) grown in CS2 medium (10–5 dilution). Histograms represent the percentage of each ComGA-EGFP localization pattern between 18 and 25 h of growth (cytoplasmic, light gray; associated with the inner face of the membrane, dark gray; single foci, black). The red curve represents the evolution of the ComGA-EGFP-expressing competent cells as a percentage of the total cells. At least 1,500 cells were counted for each time point.

As we anticipated that ComGA needed to concentrate on foci to participate in the construction of the natural transformation apparatus (22), we then wondered whether ComGA localization could be temporally and spatially dynamic. To test this hypothesis, we analyzed, along with growth, the evolution of the different localization patterns of ComGA-EGFP (Fig. 2). Indeed, when competence started to develop (after 19 h of growth), ComGA-EGFP was exclusively found diffuse in the cytoplasm of all the competent cells (Fig. 2). One hour later, cells where ComGA-EGFP localized at the inner face of the membrane started to appear. The following hour, ComGA-EGFP could be also observed as foci. Importantly, as the percentage of cells with foci increased, the percentage of cells with cytosolic or associated with the membrane localizations decreased accordingly (Fig. 2). Indeed, cells displaying a ComGA-EGFP focus represented nearly 60% of the total competent cells after 25 h of growth. This result was true for the three diluted cultures tested in which the final percentage of competent cells with a ComGA-EGFP focus was comprised between 57% and 75% (Fig. S1C).

### ComGA foci preferentially localize next to the division septum

**Fig 3:**
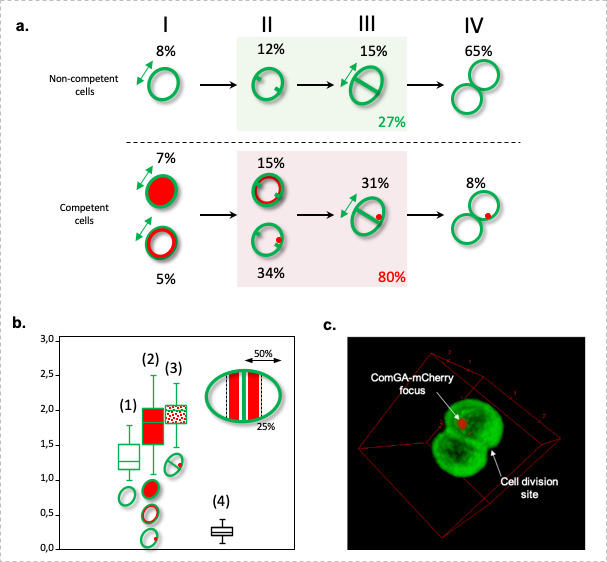
ComGA-mCherry forms foci near the division septum of *S. aureus* competent cells. (**A**) St228 (pRIT-P*_comGA_-comGA-mCh*) was grown for 25 h (10^−5^ dilution) in CS2 medium and stained with Bodipy FL Vancomycin. The percentages of non-competent and competent cells in each step of cell division are presented. In step I, single cells start elongating. Steps II and III, respectively, correspond to septum initiation and completion. Note that cell elongation is promoted in step III. Finally, daughter cell splitting occurs in step IV. Figure adapted from reference (28). (**B**) Distribution of the long cell axis length for non-competent (1) and competent (2 and 3) cells compared to the distance between ComGA foci and the septum (4). In (1), all the non-competent cells (dividing or not) have been considered. In (2), all the competent cells have been measured (dividing or not, with all the ComGA localization patterns). In (3), only the competent cells with ComGA-mCherry foci were considered. In (4) the distribution of the distance between ComGA foci and the septum of the corresponding dividing competent cell is presented. Distances were measured using the ImageJ software. In the top right corner of the graph is presented a drawing of a dividing competent cell, with the septum materialized at midcell. The red areas show the space where ComGA foci preferentially appear. (**C**) 3D reconstitution of a competent *S. aureus* cell presenting a single ComGA-mCherry focus localizing at the inner face of the membrane near the cell division site (for 360-degree rotation of this image, see supplementary video n°4).

It was previously shown that the natural transformation apparatus would localize at different cellular locations depending on the model organism considered (22, 24). Therefore, we then wondered if we could define a preferential cellular localization for the construction of the natural transformation apparatus in *S. aureus* competent cells. To reach such a goal, we constructed a *comGA-mcherry* translational fusion, expressed from the *comG* promoter, and verified that it co-localized with the ComGA-EGFP fusion (Fig. S3A). We then analyzed the localization of ComGA-mCherry, in cells for which the cell wall was stained with Vancomycin BODIPY FL (Vanco-BODIPY, Fig. 3). In this experiment, the three ComGA localization patterns, and their dynamic, could be observed when fused to mCherry (Fig. S3B). In addition, the Vanco-BODIPY staining allowed us to follow *S. aureus* cell division steps. Interestingly, recent observations have shown the morphological changes induced during the *S. aureus* cell cycle (28). These findings allowed us to correlate ComGA dynamic localization to the different stages of *S. aureus* cell division (Fig. 3).

First, we evaluated the percentage of non-competent and competent cells that were undergoing division (through the presence of a division septum, steps II and III in Fig. 3A). Under these conditions, only 27% of the non-competent cells were found with a division septum (Fig. 3A). This relatively low number was expected as the culture approaches stationary phase and the generation time increases. On the opposite, 80% of the competent cells (i.e., expressing ComGA-Mcherry) were found with an initiated or completed division septum (Fig. 3A). Interestingly, this result was confirmed by the measurement of the non-competent and competent cells’ average size. Indeed, the average length of the long axis of all the non-competent cells (dividing or not) was evaluated at 1.32 ± 0.37 µm (Fig. 3B). This average length is in accordance with previous measurements of *S. aureus* cells (28). In comparison, the average length of the long axis of all the competent cells reached 1.73 ± 0.36 µm (Fig. 3B). This result not only confirms that more competent cells are actively dividing but also indicates that dividing competent cells grow longer than dividing non-competent cells, potentially reflecting an arrest in competent cells division and an extended period of cell elongation (see step III in Fig. 3A).

Moreover, we showed that among the competent cells, cells that displayed ComGA-MCherry foci were the longest with an average length of the long axis reaching 1.93 ± 0.22 µm (Fig. 3B). This result allowed us to propose a hypothesis where ComGA dynamic localization could be coordinated with the steps of cell division. Indeed, we noticed that each ComGA localization pattern predominantly appeared at different steps of division (Fig. 3A). First, cells where ComGA-MCherry was cytoplasmic were mostly single cells (Fig. 3A). Then, cells where ComGA-MCherry was uniformly associated with the inner face of the membrane were more often single cells or cells that just initiated division (Fig. 3A). Following this dynamic, ComGA-Mcherry foci were found in dividing cells (where the division septum has been initiated or completed) and cells that just separated (Fig. 3A). Therefore, we hypothesized that localization of ComGA foci, materializing the place where natural transformation occurs, might be linked to the cell cycle.

To further investigate this last hypothesis, we finally calculated the distance between ComGA-MCherry foci and the division septum in competent cells. Interestingly, ComGA-MCherry foci were found, on average, 220 ± 90 nm away from the septum (Fig. 3B and C; Fig. S3C and Video S4). When compared to the average length of the long axis of competent cells with ComGA-MCherry foci (i.e., 1.93 µm), this result implies that the transformation apparatus is always localized in the direct vicinity of the division septum (see sketch at top right corner in Fig. 3B). Based on all these results, we provide in the discussion a model in which, during competence, cell division and natural transformation are co-regulated in space and time to ensure the completion of a horizontal gene transfer event.

### Essential natural transformation proteins co-localize with ComGA

**Fig 4:**
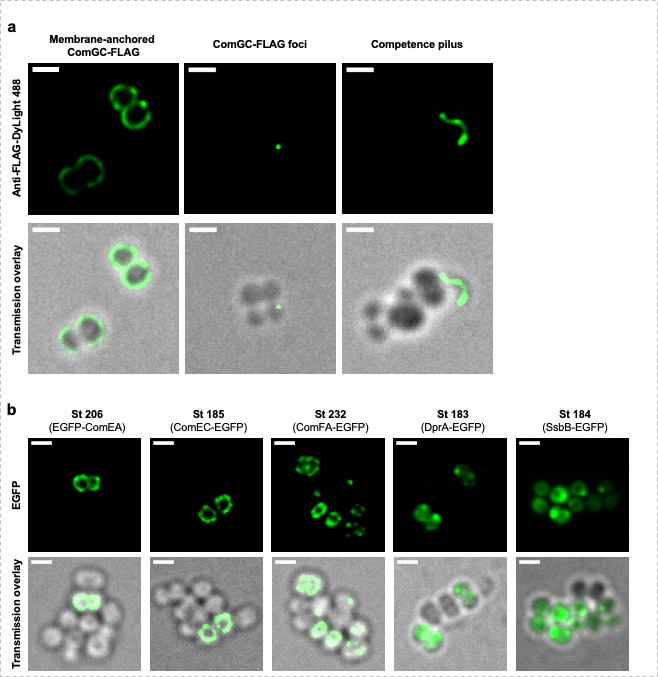
Localization of individual natural transformation proteins in *S. aureus*. (**A**) ComGC-FLAG localization patterns in strain St243 (pRIT-P*_comGA_-comGC-FLAG*) grown in CS2 medium for 25 h (10^−5^ dilution). From left to right: examples of membrane-anchored ComGC-FLAG, foci of ComGC associated with the membrane, and ComGC-FLAG forming a pilus with an average size of 0.9 ± 0.4 µm. Bar = 1 µm (**B**) Individual localization of ComEA (St206, pRIT-P*_comEA_-egfp-comEA*), ComEC (St185, pRIT-P*_comEC_-comEC-egfp*), ComFA (St232, pRIT-P*_comFA_-comEC-egfp*), DprA (St183, pRIT-P*_dprA_-dprA-egfp*), and SsbB (St184, pRIT-P*_ssbC_-ssbB-egfp*) all grown to competence for 25 h in CS2 medium (10^−5^ dilution). Proteins involved in DNA binding and uptake (ComEA, EC, and FA) all appeared associated with the membrane with ComEA and ComFA also accumulating in foci. The proteins involved in exogenous DNA processing (DprA and SsbB) were preferentially found in the cytoplasm but also accumulated in foci near the membrane. Bar = 1 µm.

Then, we decided to verify the localization of other essential actors involved in the natural transformation that were observed to co-localize with ComGA in historical model organisms (22). As the competence pilus has been shown to be essential for DNA binding and transport during natural transformation (9, 13), we first investigated its localization in competent *S. aureus* cells (Fig. 4). To do so, we inserted a FLAG tag at the C-terminus of ComGC, the major pilin. Importantly, three main types of localization (or structure) were observed (Fig. 4A). First, ComGC-FLAG was observed entirely associated with the membrane. This was not surprising, since ComGC is first anchored in the membrane and then liberated outside upon cleavage by ComC (14). In addition, in a fraction of the cells, ComGC could be observed as a single focus associated with the membrane/cell wall or as a single appendage, anchored in the membrane and extending outside of the cell (Fig. 4A). Interestingly, this extracellular structure displayed an average length of 0.9 ± 0.4 µm, a result comparable to what has been described for the pneumococcal transformation pilus (9).

We then chose to investigate the localization of proteins involved in (i) DNA transport (DNA binding, membrane-anchored ComEA protein, the plasmic membrane pore-forming protein, ComEC and the helicase-like protein, and ComFA) and (ii) DNA processing (the RecA loader, DprA, and the single strand DNA binding protein, SsbB). N- or C-terminal translational fusions of these proteins fused with EGFP were made and expressed from their natural promoter. As expected, the membrane-associated proteins, ComEA, ComEC, and ComFA, were all found localizing throughout the membrane with ComEA and ComFA also accumulating as foci near the membrane (Fig. 4B and Table S2). Furthermore, the cytosolic DNA processing proteins, DprA and SsbB, were both found diffuse in the cytosol with accumulation as foci near the membrane (Fig. 4B and Table S2). Interestingly, all the proteins examined above tend to accumulate in foci at the membrane (except ComEC), to coordinate natural transformation’s steps.

**Fig 5:**
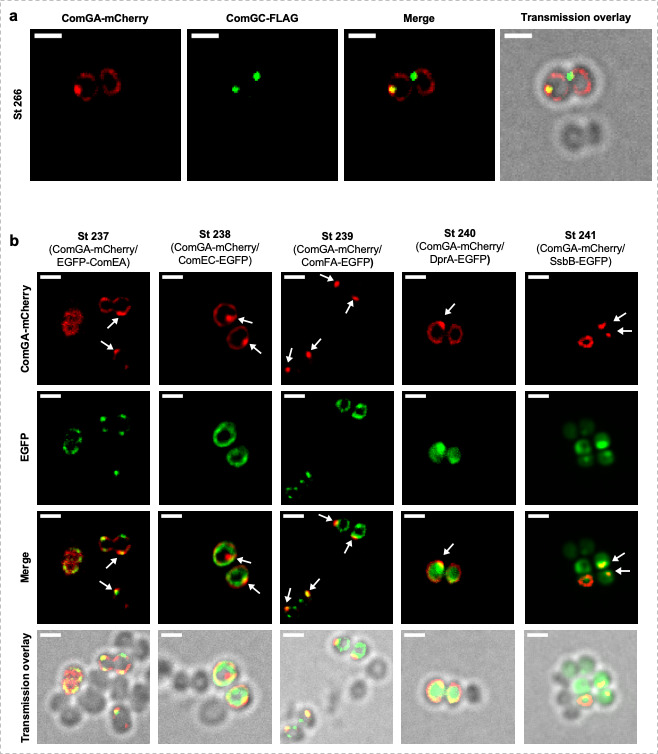
Co-localization of natural transformation proteins in competent *S. aureus* cells. (**A**) Co-localization of ComGA-mCherry and ComGC-FLAG in an overnight culture of strain St266 (pCNi-P*_comGA_-comGA-mCherry*/pRIT-P*_comGA_-comGC-FLAG*) in CS2 medium for 25 h (10^−5^ dilution). Bar = 1 µm (**B**) ComGA co-localizes with other natural transformation apparatus proteins. Foci formed by ComEA (St237, pRIT-P*_comEA_-egfp-comEA/*pCNi-PcomGA-comGA-mCh), ComFA (St239, pRIT-P*_comFA_-comEC-egfp/*pCNi-PcomGA-comGA-mCh), DprA (St240, pRIT-P*_dprA_-dprA-egfp/*pCNi-PcomGA-comGA-mCh), and SsbB (St241, pRIT-P*_ssbC_-ssbB-egfp/*pCNi-PcomGA-comGA-mCh) co-localize with ComGA-Mcherry. Only ComEC (St238, pRIT-P*_comEC_-comEC-egfp/*pCNi-PcomGA-comGA-mCh) did not show any preferential co-localization with ComGA. Arrows indicate ComGA-mCherry foci. Bar = 1 µm.

We finally analyzed the co-localization of all the proteins investigated above (i.e., ComGC, ComEA, ComEC, ComFA, DprA, and SsbB) with that of ComGA-MCherry. First, we confirmed that ComGA is involved in the transformation pilus assembly as it was found co-expressed and co-localizing with the major pilin ComGC (Fig. 5A and Table S3). This result was predominantly obtained when ComGC formed foci (Fig. 5A). Similarly, each pair of transformation proteins fused to EGFP and ComGA-MCherry was found to be co-expressed in 83%–98% of the cells, depending on the EGFP fusion considered (Table S4). Finally, we confirmed that all the transformation proteins fused to EGFP tested accumulated as foci near the membrane at the same location as ComGA-Mcherry foci (Fig. 5B), reflecting the place where natural transformation preferentially occurs. Importantly, all these protein co-localizations were confirmed by Pearson’s correlation coefficients comprised between 0.74 and 0.92 (Tables S3 and S4).

### Exogenous DNA preferentially binds in the vicinity of the transformation apparatus

**Fig 6:**
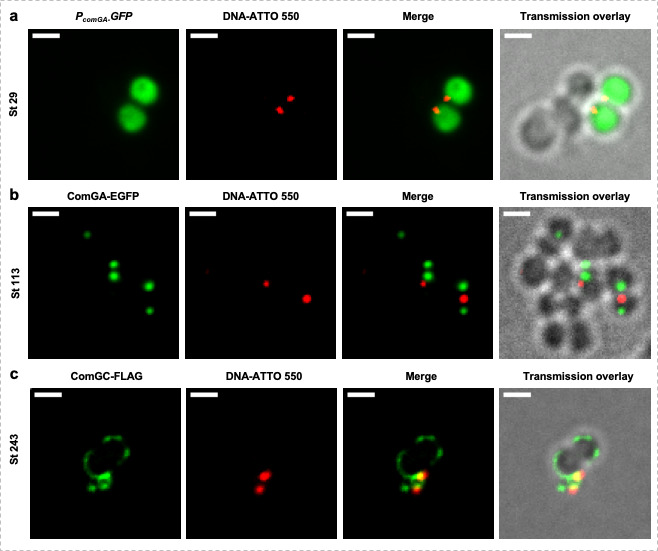
Exogenous DNA preferentially binds to *S. aureus* competent cells. (**A**) Exogenous fluorescently labeled DNA (ATTO-550) preferentially binds to competent cells from the St29 strain expressing the *gfp* gene under the control of the *comG* promoter (pRIT-P*_comG_-gfp*) grown to competence in CS2 medium for 25 h (10^−5^ dilution). Bar = 1 µm (**B**) Exogenous fluorescently labeled DNA (ATTO-550) binds to St113 competent cells (pRIT-P*_comGA_-comGA-egfp*) grown in CS2 medium for 25 h (10^−5^ dilution) in the vicinity of ComGA-EGFP foci. Bar = 1 µm (**C**) Exogenous fluorescently labeled DNA (ATTO-550) was found captured by the competence pseudopilus (containing ComGC-FLAG) stained with anti-FLAG-DyLight 488 in St243 cells (pRIT-P*_comGA_-comGC-FLAG*) grown to competence in CS2 medium for 25 h (10^−5^ dilution). Bar = 1 µm.

Finally, we wanted to visualize exogenous DNA binding at the surface of *S. aureus* competent cells. To do so, fluorescently labeled (ATTO-550-dUTP) DNA was mixed with competent cells from different strains (Fig. 6). First, using a strain expressing *egfp* under the control of the *comG* promoter (as a reporter of competence development), we confirmed that DNA binding was highly specific to competent cells (Fig. 6A and Table S5). Indeed, 82% ± 7% of the cells binding DNA were found to be competent (Table S5). Then, we particularly showed that exogenous DNA preferentially binds in the vicinity of transformasome as we evaluated that, on average, fluorescently labeled DNA would localize 300 ± 100 nm from ComGA-EGFP foci (Fig. 6C and Table S5). Finally, we observed that fluorescently labeled DNA also preferentially binds in the proximity of ComGC-FLAG (Fig. 6C and Table S5). Due to the low number of cells harboring an intact appendage, this event was not often observed. However, this result is similar to what has been observed in *S. pneumoniae* (9) and could imply that in *S. aureus*, the transformation pilus also represents the preferential exogenous DNA binding site.

## DISCUSSION

In *B. subtilis* competent cells, ComGA has been shown to first appear as diffuse in the cytoplasm and then to localize at the inner face of the membrane where it could be found nonuniformly throughout the cell with preferential accumulations at the pole(s) and/or at the septum (22). This spatial and temporal evolution of ComGA localization patterns clearly indicated that they are both dynamic and regulated. The polar accumulation of ComGA is competence dependent and therefore candidates interacting with ComGA and directing it to specific cellular localizations must be the product of a late competence gene (22). Indeed, it has been proposed that ComEB, the second gene of the *comE* operon encoding a dCMP deaminase, could be the mediator for ComGA polar localization in *B. subtilis* (29). Importantly, we observed the same kind of spatial and temporal dynamic in *S. aureus*. Therefore, it is also tempting to propose that ComGA localization in *S. aureus* could also be directed by a late competence protein. However, because ComGA displays a polar localization in *B. subtilis*, while it tends to accumulate next to the division site in *S. aureus*, the ComGA localization mediator might be different in these two model organisms and further studies will be required to identify ComGA anchor in *S. aureus*.

Our observations also support the idea of the presence of multi-protein machinery, assembled at a unique cellular localization to perform binding, transport, and processing of transforming DNA. The individual proteins we have studied here are localized in similar patterns (except ComEC which did not form foci), and apparent proximity when tagged with distinct fluorophores. ComGA (a traffic ATPase needed for dsDNA binding and required for the competence pilus assembly), co-localizes with ComGC (the major competence pilus’ pilin), ComEA (a dsDNA binding protein), ComFA (a protein required for DNA transport), and DprA or Ssb (two proteins involved in ssDNA processing). Importantly, while ComGC and ComEA are both exposed outside in the cell wall, ComGA and ComFA are predicted to be associated with the inner face of the membrane, and DprA or Ssb supposed to be cytosolic. Therefore, proteins required at different steps during natural transformation and targeted to different cellular compartments were found to be co-localize in competent cells. These observations suggest that binding, uptake, and processing of transforming DNA are closely coordinated processes, in space and time, involving multiple protein-protein and DNA-protein interactions. Several reports have already shown that this was true in various model organisms and it will be essential to investigate this field in *S. aureus* in the future.

In addition, several natural transformation protein foci for an estimated 50 uptake sites per competent cell have been proposed in *B. subtilis*, suggesting that each of the fluorescent focus may contain several uptake machinery (30). This might also be true in *S. aureus*. However, the fact that almost all *S. aureus* competent cells displayed only one focus of transformation proteins might indicate that fewer uptake machinery might be assembled than in *B. subtilis*, potentially explaining, at least partially, the lower transformation efficiencies observed in this human pathogen (7).

Very interestingly, our results also show that ComGA preferentially localizes in the vicinity of *S. aureus* competent cells division septum (while most non-competent cells are not dividing). In addition, we establish a correlation between the dynamic of ComGA localization and the cell cycle stages. Therefore, we propose a model in which the natural transformation apparatus’ localization, in the direct vicinity of the competent cell septum has a regulatory function in *S. aureus*: to establish a spatial and temporal link between DNA binding/uptake/processing and cell division.

In such model, competence-inducing *S. aureus* cells express ComGA which first appears diffuse in the cytoplasm (step I in Fig. 3A). As ComGA starts to uniformly associate with the inner face of the membrane, competent cells initiate division (step II in Fig. 3A). The initiation of the septum would then represent a spatial reference for ComGA accumulation, materializing the place where the transformation apparatus is established and natural transformation occurs (steps II and III in Fig. 3A). Finally, cell division completion generates two daughter cells with only one of them harboring a ComGA focus (step IV in Fig. 3A).

Finally, division inhibition during genetic competence for natural transformation has been demonstrated in several model organisms. For example, the early competence protein ComM delays division in the Gram-positive coccus, *S. pneumoniae* (25). The authors proposed that all these mechanisms could ensure natural transformation completion before the resumption of cell division.

Interestingly, it has been shown that during step III of division, *S. aureus* cells presenting a completed septum tend to elongate before septum constriction and daughter cell separation (28). If septum synthesis and/or constriction is postponed in competent cells during steps II and III (during which all the proteins co-localize to form the transformation apparatus), cell elongation could be prolonged, leading to longer competent cells and providing time for natural transformation completion. Interestingly, initiation of constriction of the cytokinetic ring and completion of cell division have been shown to be delayed by ComM during natural transformation in *S. pneumoniae* (25). It will be important in the future to identify an early competence protein, similar to ComM from *S. pneumoniae*, regulating the cell cycle during natural transformation in *S. aureus* competent cells.

## MATERIALS AND METHODS

### Bacterial strains and growth conditions

All bacterial strains and plasmids used in this study are listed in Table S6. *S. aureus* strains were grown at 37°C under aerobic conditions in brain heart infusion (BHI) or competence-inducing synthetic medium (CS2). CS2 was freshly prepared from stock solutions as previously described (4).

*Escherichia coli* IM08B or DH10B strains were used as vector hosts to amplify plasmids and were cultured in Luria-Bertani (LB) medium at 37°C under aerobic conditions. When needed, one or several of the following antibiotics were added to the culture media depending on the bacterial genotype: 100 µg/mL ampicillin (Amp), 10 µg/mL chloramphenicol (Cm), 200 µg/mL kanamycin (Kan), and 50 µg/mL neomycin (Neo).

### Plasmids and strains construction

Briefly, all the late competence genes studied in this work were amplified from the chromosomal DNA of *S. aureus* N315 ex w/o ϕ (St12) with their respective native promoters. Table S7 presents more details about these genes and their promoters. Staphylococcal genomic DNA was extracted using the NucleoSpin Microbial DNA extraction kit (Macherey-Nagel) according to the producer’s protocol. Promoters, genes, and linear plasmid vectors were amplified by PCR using the Phusion High-Fidelity DNA Polymerase (Thermo Scientific) and the appropriate primers following the manufacturer’s recommendations. A list of all the primers used in this work is detailed in Table S8. PCR products were checked by electrophoresis onto 1% agarose gel and purified by NucleoSpin PCR and gel clean-up kit (Machery-Nagel). Linear DNA fragments (inserts and vectors) with 3′ and 5′ overlapping regions were then assembled with a ratio of 1:3 (vector to insert) using the Gibson method with a homemade mixture containing 0.005 U/µL T5 Exonuclease, 0.03 U/µL Phusion High-Fidelity DNA Polymerase, and 5 U/µL T4 DNA Ligase in ISO buffer (5% wt/vol PEG-8000, 100 mM Tris HCl, pH 7.5, 10 mM MgCl2, 10 mM DTT, 1 mM NAD, and 1 mM each dNTP). All reagents used for Gibson assembly were purchased from New England BioLabs. The reaction was performed in a total volume of 20 µL for 1 h at 50°C then inactivated by incubation for 10 min at 80°C. An amount of 5 µL of the Gibson assembly products was electroporated into 45 µL of electrocompetent *E. coli* IM08B (2.5 kV, 1 ms). Positive clones were selected on LB-Amp agar plates and checked by colony-PCR using DreamTaq Green PCR Master Mix (Thermo Scientific) with the appropriate primers. Plasmids from selected positive clones were extracted and purified on silica columns (NucleoSpin Plasmid, Macherey-Nagel), then sequenced with their specific oligonucleotides (Eurofins Genomics). Finally, 500 ng of plasmids was used for transfer in electrocompetent *S. aureus* cells (1.8 kV, 2.5 ms). Transformed clones were selected on BHI agar plates with the appropriate antibiotics and checked by colony-PCR. Positive clones were cultured overnight and stored at −80°C in sterile cryogenic vials with 16% sterile glycerol.

#### pRIT-P*_comGA_-comGA-egfp*

It is important to mention that the expression of the *comGA-egfp* translational fusion was performed from the low-copy number pRIT plasmid as no signal could be observed when expressed as a single copy on the chromosome. As *comGA* is one of the most induced genes during competence (7, 31, 32), we also expressed the other translational fusions from the pRIT or pBCB low-copy number vectors.

To construct the plasmid pRIT-P*_comGA_-comGA-egfp*, a 1,228 bp DNA fragment was first amplified from purified genomic DNA of N315 ex w/o ϕ (St 12) using the primers P152 and P220. This sequence contained the native promoter P*_comGA_* (256 bp) and the *comGA* gene without the stop codon (972 bp) flanked by KpnI restriction sites at both 5′ and 3′ termini. Purified KpnI-digested inserts were then ligated into the KpnI-digested pBCB-8-GK plasmid and electroporated into *E. coli* IM08B. The pBCB-P*_comGA_-comGA-egfp* plasmid was purified, verified by PCR, and sequenced to ensure the correct orientation of the insert and the absence of mutations.

The P*_comGA_-comGA-egfp* DNA fragment was amplified from the pBCB-P*_comGA_-comGA-egfp* using primers P320 and P321 and assembled using Gibson’s technique with the pRIT vector amplified using the primers P322 and P323. The resulting Gibson assembly was introduced into electrocompetent IM08B cells, and positive clones were selected on LB-Amp agar plates and checked by colony-PCR using the primers P335 and P336. The plasmid was finally extracted, purified, and sequenced before electroporation into St 12 electrocompetent cells, thus creating the strain St113.

#### Other pRIT-P_com_-*com-egfp*

pRIT-P*_comGA_-comGA-egfp* is a low-copy-number *E. coli-S. aureus* shuttle plasmid that allows the expression of ComGA under the control of its native promoter with a C-terminal EGFP translational fusion, mediated by a flexible 13 amino-acids linker. This plasmid served as an original template for the construction of all other pRIT plasmids used in this study using the same methodology except for the plasmid pRIT-P*_comEA_-egfp-comEA* in which the *egfp* translational fusion was designed at the N-terminal extremity of the *comEA* gene, separated by a 10 amino-acids linker.

#### pRIT-P*_comGA_-comGA-mCh*

With the aim of co-localizing ComGA-mCherry with other EGFP-fused late competence genes, we first designed the high-copy-number *S. aureus* replicative plasmid pCNi by replacing the erythromycin resistance cassette *ermC* of the plasmid pCN35 with the kanamycin resistance cassette *aphA-3* from the plasmid pCN34 using ApaI and SacII restriction enzymes (FastDigest, Thermo Scientific). The resulting pCNi plasmid contained the staphylococcal origin of replication pT181*cop*-623 *repC* and the kanamycin resistance cassette *aphA-3*. pCNi was first produced in DH10B cells, then transferred into RN 4220 and finally into St12 thus creating the strain St224. Using the Gibson assembly technique, we created the plasmid pRIT-P*_comGA_-comGA-mCh* by replacing *egfp* with the Mcherry sequence. Finally, the P*_comGA_-comGA-mCh* DNA fragment was amplified from the pRIT-P*_comGA_-comGA-mCh* and assembled using Gibson’s technique with the pCNi vector, producing the pCNi-P*_comGA_-comGA-mCh*.

### Natural competence development

The desired *S. aureus* strains were inoculated onto BHI agar plates, with the appropriate antibiotics if needed, and incubated at 37°C for 24 h. CS2 suspensions adjusted to OD_600 nm_ of 0.5 were prepared from liquid BHI pre-cultures in the exponential phase of growth. Serial 10-fold dilutions were then prepared in a total volume of 10 mL in an individual sterile 50 mL Falcon tube and incubated overnight at 37°C with shaking at 120 rpm. Cell density was monitored hourly, and 500 µL of samples was collected in sterile Eppendorf tubes. Cell pellets were washed with sterile phosphate-buffered saline (PBS), fixed with 1.6% formaldehyde (Invitrogen) for 20 min at room temperature, rinsed twice in equal volumes of sterile PBS, and finally re-suspended in 100 µL of 45% glycerol buffer for storage at −20°C.

### Spinning disk microscopy

An amount of 10 µL of fixed cells was mounted on a 1% agarose-coated slide with a drop of mounting medium (Fluoromount-G). Slides were then observed under a confocal microscope Nikon Eclipse Ti-E equipped with a spinning disk module (Yokogawa CSU-X1-A1) and a super-resolution system (Live SR 3D, Gataka systems) equipped with a ×100 oil immersion objective (Nikon; NA = 1.49). Fluorescence images were recorded using the 470/30 nm excitation filter and 520/35 nm filter for GFP, and the 560/40 nm excitation filter and 630/60 nm filter for mCherry. Images were processed with the Metamorph software (Molecular Devices). Images were finally analyzed with the FiJi software (ImageJ 1.53c).

### Vancomycin, BODIPY FL Conjugate (VMB) labeling

Cells were labeled with Vancomycin BODIPY FL (Vanco-BODIPY, Life Technologies) at a final concentration of 2 µg/mL for 30 min at 37°C. Excess incorporated labeling was removed by washing the cells three times in sterile PBS and finally fixed as explained above.

### Competence pseudopilus visualization

To observe the staphylococcal competence pseudopilus, 1 mL of overnight cultures in CS2 of strains expressing ComGC-FLAG was collected in a sterile Eppendorf tube, centrifuged at 4,000 *g* for 3 min, and washed with sterile PBS. Cells were fixed directly on SuperFrost Plus slides with 3.2% paraformaldehyde (PFA) and labeled with anti-FLAG-DyLight 488 (1:200, ThermoFisher).

### Fluorescent DNA preparation

For DNA binding experiments, a 1 kb fluorescent DNA was first synthesized by PCR using Aminoallyl-dUTP-ATTO-550 (JenaBioscience). Briefly, we used 1 µL of Extaq enzyme from Takara on genomic DNA (20 µg) with 1 µL each primer, 0.25 µM of each dNTP, and 0.1 µM of ATTO-550 dUTP. Elongation was performed at 72°C for 3 min per kb, fragments were purified (Machery-Nagel) and assessed by spectrophotometry to check the incorporation of fluorescent nucleotides.

In all, 250 µL of overnight *S. aureus* cultures in CS2 was collected into a sterile tube, washed, resuspended in an equal volume of filtered CS2, preheated to 37°C, and containing 500 ng of fluorescent DNA and incubated for 5 min at 37°C with gentle shaking. Samples were finally fixed as mentioned above.

### Data analysis of microscopy images

The ImageJ-based Coloc2 plug-in was employed to determine Pearson’s correlation coefficient (PCC). Individual cells were cropped out to remove the low-intensity and highly correlated background in both channels. The PCC values presented are an average of at least 10 individual cells for each protein couple. The PCC is a well-established measure of correlation and has a range of +1 (perfect correlation) to −1 (perfect but negative correlation), with 0 denoting the absence of a relationship. In each case, the absence of fluorescence emission bleed-through was verified using individual fusions.

## SUPPLEMENTAL MATERIAL

Supplemental material spectrum.02807-23-s0001.pdf Fig. S1 to S3; Tables S1 to S8.

Supplemental legends spectrum.02807-23-s0002.pdf Legends for Videos S1 to S4.

Video S1 spectrum.02807-23-s0003.avi 360° rotation of an S. aureus competent cell with cytoplasmic ComGA-EGFP.

Video S2 spectrum.02807-23-s0004.avi 360° rotation of an S. aureus competent cell with ComGA-EGFP associated to the inner face of the membrane.

Video S3 spectrum.02807-23-s0005.avi 360° rotation of an S. aureus competent cell with a ComGA-EGFP focus.

Video S4 spectrum.02807-23-s0006.avi 360° rotation of an S. aureus competent cell harboring a ComGA-mCherry focus and stained with Vanco-BODIPY.

ASM does not own the copyrights to Supplemental Material that may be linked to, or accessed through, an article. The authors have granted ASM a non-exclusive, world-wide license to publish the Supplemental Material files. Please contact the corresponding author directly for reuse.
