# Supplementary material for: Natural transformation and cell division delay in competent Staphylococcus aureus
Source: Microbiol Spectr. 2023 Oct 13;11(6):e02807-23. doi: 10.1128/spectrum.02807-23 (PMC10714784; doi:10.1128/spectrum.02807-23)
Supplement: Supplemental material — Fig. S1 to S3; Tables S1 to S8. [file spectrum.02807-23-s0001.pdf]

Supplementary Information

Genetic transformation and cell division delay in competent *Staphylococcus aureus*

Fedy Morgene [1], Célia Rizoug Zeghlache [1], Shi Yuan Feng[1], Yolande Hauck[1] and Nicolas Mirouze [*,1]

[*] Corresponding author
[1] Université Paris-Saclay, CEA, CNRS, Institute for Integrative Biology of the Cell (I2BC), 91198, Gif-sur-Yvette, France

This PDF file includes:

Supplementary figures 1 to 3
Supplementary tables 1 to 8
Supplementary videos 1 to 4

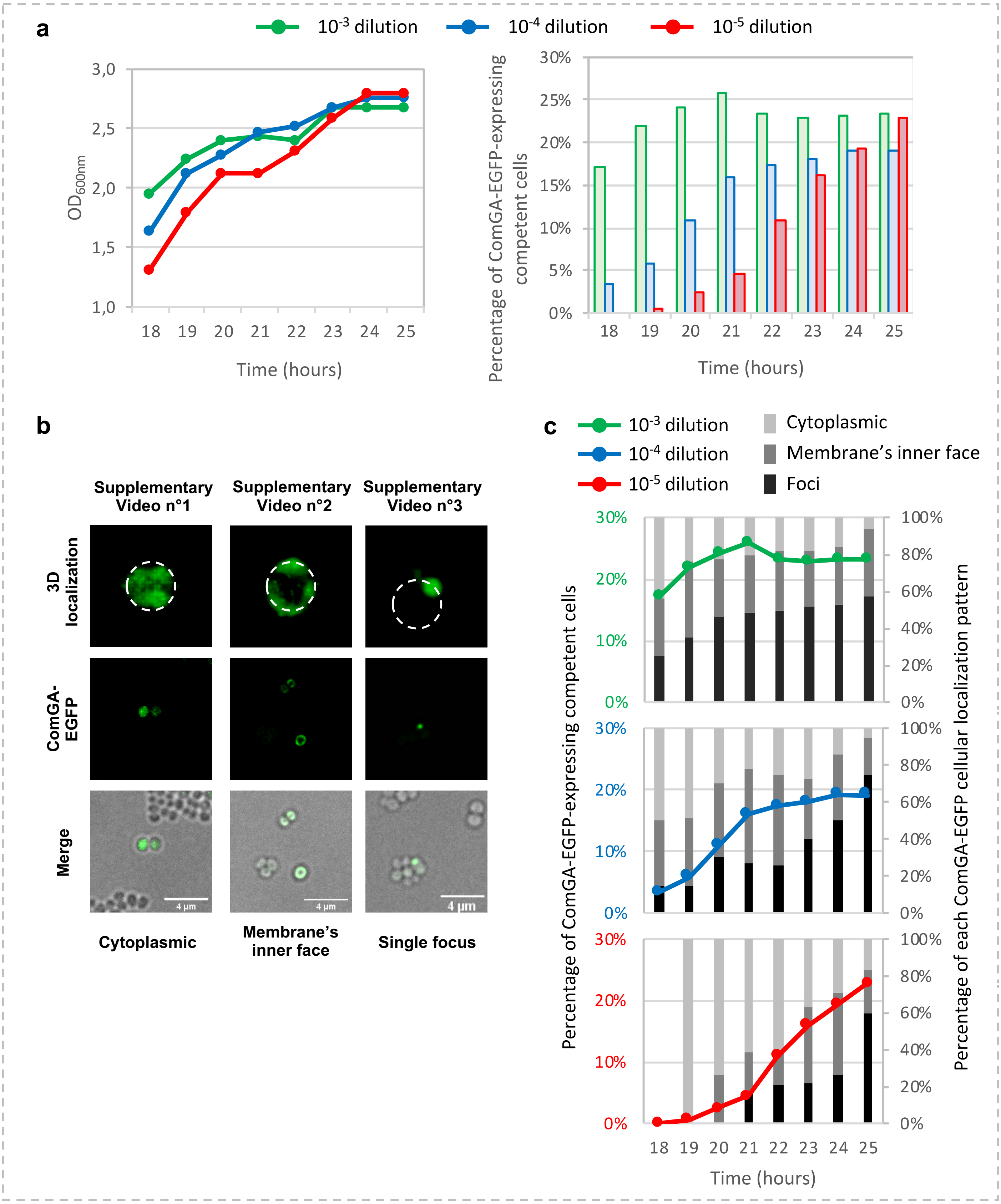

Supplementary Fig. 1. Spatial and temporal dynamic of ComGA-EGFP localization in competent *S. aureus* cells.

(a) Evolution of ComGA-EGFP-expressing cells as a percentage of total cells. Samples from serial 10-fold dilutions (10^{-3} in green, 10^{-4} in blue and 10^{-5} in red) of strain St113 (pRIT-P$_{comGA}$-*comGA-egfp*) grown in CS2 medium (left panel) were collected and analyzed microscopically for ComGA-EGFP expression (right panel).

(b) Examples of the three ComGA-EGFP (pRIT-P$_{comGA}$-*comGA-egfp*) cellular localizations observed using the St113 strain grown in CS2 medium (from left to right: cytoplasmic, associated to the inner face of the membrane and accumulation in foci near the membrane).

For each localization pattern, we provide 3D reconstructions (top row) as well as 360 degrees videos.

Bar = 4 µm

(c) Dynamic of ComGA-EGFP localization patterns in *S. aureus* competent cells from diluted cultures (10^{-3} top panel; 10^{-4}, middle panel; 10^{-5}, bottom panel). In addition to the evolution of the percentage of ComGA-EGFP-expressing cells (10^{-3} in green, 10^{-4} in blue and 10^{-5} in red), histograms represent the percentage of each ComGA-EGFP localizing pattern : in the cytoplasm (light grey), associated to the membrane (dark grey) or as single foci (black). At least 1500 cells were counted for each time point in each culture.

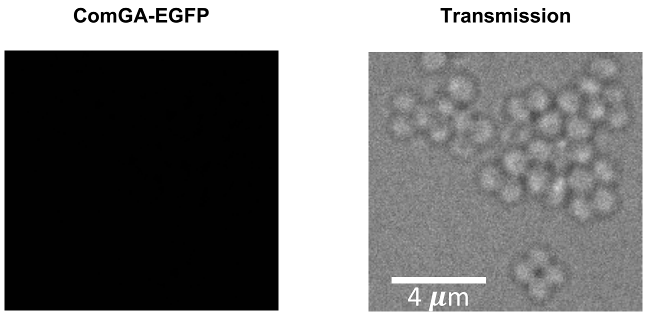

Supplementary Fig. 2. Expression of P_{comGA}-*comGA-egfp* from the pRIT is still dependent on SigH. Strain St116 (Δ*sigH*, pRIT-P_{comGA}-*comGA-egfp*) grown in CS2 medium (dilution 10^{-3} for 24 hours) does not show any expression of *comGA-egfp* from the native P_{comGA} promoter present on the pRIT in the absence of SigH.
Bar = 4 μm

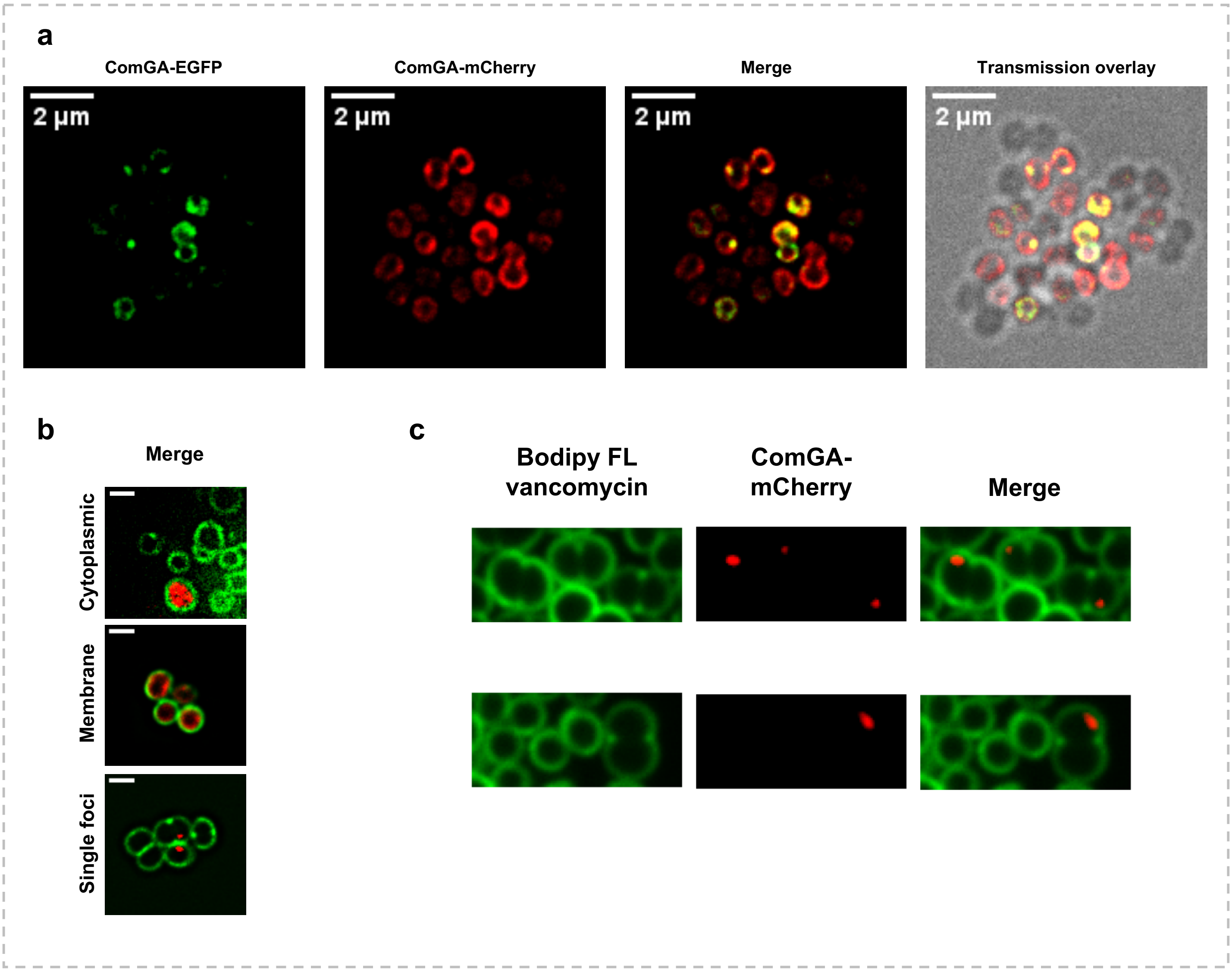

Supplementary Fig. 3. ComGA-mCherry localization in competent *S. aureus* cells.

(a) Samples from strain St242 (pRIT-P_{comGA}-*comGA-egfp* / pCNi-P_{comGA}-*comGA-mCherry*) grown in CS2 medium (dilution 10^{-3} for 24 hours) show co-localization of the two fusion proteins.

Bar = 2 µm

(b) Examples of the three localizations of ComGA-Mcherry (cytoplasmic, top panel; associated to the inner face of the membrane, middle panel and single foci, bottom panel) in *S. aureus* competent cells (St228, *pCNi-*P_{comGA}*-comGA-mCh*) stained with Bodipy FL Vancomycin.

Bar = 1 µm

(c) Examples of ComGA-MCherry (St228, *pCNi-*P_{comGA}*-comGA-mCh*) foci localizing next to the initiated septum in *S. aureus* competent cells.

Supplementary Table 1. ComGA-EGFP localization in *S. aureus* competent cells.

Total counted cells	% of cells with ComGA-EGFP	% of cells with ComGA foci	% of cells with a single ComGA focus	% of cells with two ComGA foci	% of cells with more than two ComGA foci
13664	1548 (11.3%)	478 (31.0%)	474 (99.2%)	3 (0.6%)	1 (0.2%)

Strain St113 (pRIT-P$_{comGA}$-*comGA-egfp*) was grown to competence for 24h in CS2 medium (10^{-3} dilution). The percentage of ComGA-EGFP-expressing cells and among those the percentage of cells with ComGA-EGFP foci were evaluated by microscopy. Finally, among the cells presenting ComGA-EGFP foci, the percentage of cells with one, two or more foci was calculated.

This table completes the results presented in Fig. 1.

At least 400 cells were counted for each strain.

Supplementary Table 2. Statistics for ComEA, ComEC, ComFA, DprA and SsbB localization in competent *S. aureus* cells.

**Strain**	**St 206**	**St 185**	**St 232**	**St 183**	**St 184**
EGFP translational fusion	EGFP-ComEA	ComEC-EGFP	ComFA-EGFP	DprA-EGFP	SsbB-EGFP
Total EGFP+ %	18%	9%	36%	30%	63%
Cytoplasmic	0%	0%	0%	69%	45%
Membrane	55%	90%	85%	0%	0%
Membrane foci	45%	10%	15%	31%	55%

Strains St206 (pRIT-P$_{comEA}$-*egfp-comEA*), St185 (pRIT-P$_{comEC}$-*comEC-egfp*), St232 (pRIT-P$_{comFA}$-*comEC-egfp*), St183 (pRIT-P$_{dprA}$-*dprA-egfp*) and St184 (pRIT-P$_{ssbC}$-*ssbB-egfp*) were grown to competence for 25h in CS2 medium (10^{-5} dilution). The percentage of cells expressing each EGFP fusion as well as the percentage of each localization pattern were evaluated by microscopy.

This table completes the results presented in Fig. 5.

At least 400 cells were counted for each strain.

Supplementary Table 3. ComGC-FLAG localization in *S. aureus* competent cells.

Strain	Counted cells	%ComGA	%ComGC	% ComGC+ in ComGA	% ComGA+ in ComGC	Pearson's correlation coefficient
St 266	474	42%	28%	56%	85%	0.88 ± 0.08

Strain St266 (pCNi-P$_{comGA}$-*comGA-mCherry* / pRIT-P$_{comGA}$-*comGC-FLAG*) was grown to competence for 25h in CS2 medium (10^{-5} dilution). The percentage of ComGA-mCherry-expressing cells, ComGC-FLAG-expressing cells as well as the percentage of ComGA-mCherry-expressing cells that also express ComGC-FLAG and ComGC-FLAG-expressing cells that also express ComGA-mCherry were evaluated by microscopy. Pearson's correlation coefficients were calculated based on 10 individual cells as explained in the Material and methods.

This table completes the results presented in Fig. 4.

At least 400 cells were counted for each strain.

Supplementary Table 4. ComEA, ComEC, ComFA, DprA and SsbB co-expression and co-localization with ComGA-Mcherry in *S. aureus* competent cells.

Strain	St 237	St 238	St 239	St 240	St 241	St 242
EGFP translational fusion	EGFP-ComEA	ComEC-EGFP	ComFA-EGFP	DprA-EGFP	SsbB-EGFP	ComGA-EGFP
Total counted cells	415	429	438	531	424	417
% of ComGA-mCherry-expressing cells	32%	50%	39%	47%	31%	48%
% of EGFP-expressing cells	17%	41%	33%	34%	60%	34%
% of cells co-expressing EGFP fusions in ComGA-mCherry-expressing cells	46%	78%	79%	69%	**98%**	69%
% of cells co-expressing ComGA-mCherry in EGFP-expressing cells	**83%**	**95%**	**92%**	**94%**	51%	**98%**
Pearson's correlation coefficient	**0.74 ± 0.08**	**0.74 ± 0.13**	**0.82 ± 0.13**	**0.84 ± 0.09**	**0.84 ± 0.05**	**0.92 ± 0.06**

Strains St237 (pRIT-P_{comEA}-*egfp-comEA / pCNi-*P_{comGA}*-comGA-mCh*), St238 (pRIT-P_{comEC}-*comEC-egfp / pCNi-*P_{comGA}*-comGA-mCh*), St239 (pRIT-P_{comFA}-*comEC-egfp* / pCNi-PcomGA-comGA-mCh), St240 (pRIT-P_{dprA}-*dprA-egfp / pCNi-*P_{comGA}*-comGA-mCh*), St241 (pRIT-P_{ssbC}-*ssbB-egfp / pCNi-*P_{comGA}*-comGA-mCh*) and St242 (pRIT-P_{comGA}-*comGA-egfp / pCNi-*P_{comGA}*-comGA-mCh*) were grown to competence for 25h in CS2 medium (10^{-5} dilution). The percentage of cells expressing ComGA-Mcherry, expressing each EGFP fusion as well as the percentage of ComGA-mCherry-expressing cells that also express the EFGP fusion and EGFP fusion-expressing cells that also express ComGA-mCherry were evaluated by microscopy. At least 400 cells were counted for each strain.

Finally, for each EGFP fusion co-expressed with ComGA-MCherry, the Pearson's correlation coefficient (PCC) was calculated based on 10 individual cells as explained in the Material and methods. This table completes the results presented in Fig. 5.

Supplementary Table 5. Exogenous DNA binding at the surface of *S. aureus* competent cells.

Strain	Counted cells	%GFP	%DNA	%GFP+ in DNA+	%DNA+ in GFP+
St29	408	48%	14%	**82%**	23%

Strain	Counted cells	%ComGA-EGFP	%DNA	%GFP+ in DNA+	%DNA+ in ComGA-EGFP+
St113	401	31%	12%	**80%**	31%

Strain	Counted cells	%ComGC-FLAG+	%DNA	%FLAG+ in DNA+	%DNA+ in ComGC-FLAG+
St243	427	23%	10%	**88%**	38%

Strains St29 (pRIT-P$_{comGA}$-*egfp*), St113 (pRIT-P$_{comGA}$-*comGA-egfp*) and St243 (*pRIT-PcomGA-comGC-flag*) were grown to competence for 23h in CS2 medium (10^{-5} dilution). After exposition to fluorescently labelled DNA, the percentage of competent cells, of cells binding DNA were evaluated. In addition, the percentage of cells binding DNA that were also competent as well as competent cells that were also binding DNA was calculated (two last columns).

This table completes the results presented in Fig. 6.

At least 400 cells were counted for each strain.

Supplementary table 6. Bacterial strains and plasmids

Bacteria strains				
**Strain**	**Genotype / plasmid**	**Phenotype**	**Parental**	**Source**
Escherichia coli				
**IM08B**	Δ*dcm (*does not methylate DNA on cytosine*)* ΩP$_{help}$-*hsdMS* ΩP$_{N25}$-*hsdS* (type 1 modification gene from *S. aureus* CC8-2 and CC8-1, respectively)	Stp^R	DC10B	(1)
**DH10B**	F$^-$ Δ*mcr*A ϕ80*lac*ZΔM15 Δ*lac*X74 Δ*rec*A1 Δ*end*A1 *ara*D139 Δ(*ara leu*) λ$^-$	Stp^R	K-12	(2)
Staphylococcus aureus				
**St 12**	N315 ex w/o ϕ (lacking *SCCmec* and cured from phages)	Erm^R	N315	(3)
**St 29**	N315 ex w/o ϕ / pRIT-P$_{comGA}$-*gfp*	Erm^R, Cm^R	St 12	(3)
**St 113**	N315 ex w/o ϕ / pRIT-P$_{comGA}$-*comGA-egfp*	Erm^R, Cm^R	St 12	This study
**St 116**	N315 ex w/o ϕ Δ*sigH*/ pRIT-P$_{comGA}$-*comGA-egfp*	Erm^R, Cm^R	St 113	This study
**St 183**	N315 ex w/o ϕ / pRIT-P$_{dprA}$-*dprA-egfp*	Erm^R, Cm^R	St 12	This study
**St 184**	N315 ex w/o ϕ / pRIT-P$_{ssbC}$-*ssbB-egfp*	Erm^R, Cm^R	St 12	This study
**St 185**	N315 ex w/o ϕ / pRIT-P$_{comEC}$-*comEC-egfp*	Erm^R, Cm^R	St 12	This study
**St 206**	N315 ex w/o ϕ / pRIT-P$_{comEA}$-*egfp-comEA*	Erm^R, Cm^R	St 12	This study
**St 228**	N315 ex w/o ϕ / pCNi-P$_{comGA}$-*comGA-mCh*	Erm^R, Kan^R	St 12	This study
**St 232**	N315 ex w/o ϕ / pRIT-P$_{comFA}$2-*comFA-egfp*	Erm^R, Cm^R	St 12	This study
**St 237**	N315 ex w/o ϕ / pCNi-P$_{comGA}$-*comGA-mCh* / pRIT-P$_{comEA}$-*comEA-egfp*	Erm^R, Cm^R, Kan^R	St228	This study
**St 238**	N315 ex w/o ϕ / pCNi-P$_{comG}$-*comGA-mCh* / pRIT-P$_{comEC}$-*comEC-egfp*	Erm^R, Cm^R, Kan^R	St228	This study
**St 239**	N315 ex w/o ϕ / pCNi -P$_{comGA}$-*comGA-mCh* / pRIT-P$_{comFA}$-*comFA-egfp*	Erm^R, Cm^R, Kan^R	St228	This study
**St 240**	N315 ex w/o ϕ / pCNi -P$_{comGA}$-*comGA-mCh* / pRIT-P$_{dprA}$-*dprA-egfp*	Erm^R, Cm^R, Kan^R	St228	This study
**St 241**	N315 ex w/o ϕ / pCNi -P$_{comGA}$-*comGA-mCh* / pRIT-P$_{ssbC}$-*ssbB-egfp*	Erm^R, Cm^R, Kan^R	St228	This study
**St 242**	N315 ex w/o ϕ / pCNi -P$_{comGA}$-*comGA-mCh* / pRIT-P$_{comGA}$-*comGA-egfp*	Erm^R, Cm^R, Kan^R	St228	This study
**St 243**	N315 ex w/o ϕ / pRIT-P$_{comGA}$-*comGC-FLAG*	Erm^R, Cm^R	St 12	This study
**St 266**	N315 ex w/o ϕ / pCNi -P$_{comGA}$-*comGA-mCh* / pRIT-P$_{comGA}$-*comGC-FLAG*	Erm^R, Cm^R, Kan^R	St 228	This study

Plasmids		
**Name**	**Description**	**Source**
**pRIT-P$_{comGA}$-*gfp***	*E. coli – S. aureus* low-copy-number shuttle vector (*AmpR – CmR*). Expression of GFP under the control of comGA promoter (PcomGA).	(3)
**pBCB-7-ChK**	Non-replicative plasmid (*AmpR – KanR).* Encoding mCherry gene flanked by C and N terminal linkers.	(4)
**pBCB-8-GK**	Non-replicative plasmid (*AmpR – KanR).* Encoding EGFP gene flanked by C and N terminal linkers.	(4)
**pCN34**	*E. coli – S. aureus* low-copy-number shuttle vector (*AmpR – KanR)*	(5)
**pCN35**	*E. coli – S. aureus* high-copy-number shuttle vector (*AmpR – ErmR)*	(5)

Supplementary table 7. *S. aureus* late competence genes studied

Gene	Locus tag	Gene length	Promoter	Promoter length
comGA	SA1374	975 bp	P_{comGA}	256 bp
comGC	SA1372	312 bp	P_{comGA}	256 bp
comEA	SA1418	687 bp	P_{comEA}	284 bp
comEC	SA1416	2202 bp	P_{comEC}	224 bp
comFA	SA0705	1083 bp	P_{comFA}	1815 bp
dprA	SA1092	873 bp	P_{dprA}	325 bp
ssbC	SA1899	396 bp	P_{ssbC}	193 bp

Supplementary table 8. Primers used in this study

Primer	Name	5' oligonucleotide sequence	Amplified DNA	DNA template	Used to
P152	KpnI-P_{comGA}-F	GCA GGT ACC GTT CGA TGA ATT CGC AGT TGT TGG AGA TAC	P_{comGA}-*comGA*	N315 genomic DNA	Create pBCB-P_{comGA}-*comGA-egfp* by ligating KpnI-digested P_{comGA}-*comGA* insert into KpnI-digested pBCB8-GK plasmid
P220	KpnI-*comGA*-R	GCA GGT ACC AAT GTA TTT ATC CAT TGT AGT TTC ACA AAT GAC ACC TGC			
P320	pBCB-*comGA*-F	GGA GGT GTT TTT TTG AAG ATT CTA TTT CAA GAA ATA ATT AAT AAA GCG	P_{comGA}-*comGA-egfp*	pBCB-P_{comGA}-*comGA-egfp*	Create pRIT-P_{comGA}-*comGA-egfp* by Gibson assembly
P321	pRIT-*egfp*-SpeI-R	GCT ATG ACC ATG ATT ACG CCA AGC TGC ACT AGT GCT CAG CCG			
P322	PstI-pRIT-F	CGG CTG AGC ACT AGT GCA GCT TGG CGT AAT CAT GGT CAT AGC	pRIT-P_{comGA}	pRIT-P_{comGA}-*gfp*	
P323	pRIT-P_{comGA}-R	CGC TTT ATT AAT TAT TTC TTG AAA TAG AAT CTT CAA AAA AAC ACC TCC			
P335	pRIT-X-F	GGC TTA ACT ATG CGG CAT CAG AGC	Variable	All pRIT plasmids	Check insertions in pRIT plasmids
P336	pRIT-X-R	GAA TTG TGA GCG GAT AAC AAT TTC ACA CAG G			
P376	LinkerA-*egfp*-F	GGT ACC AGC GCT ATC GAT CGG CCG	pRIT-*egfp*	pRIT-P_{comGA}-*comGA-egfp*	Amplify pRIT-*egfp* linear plasmid fragment for Gibson assembly
P377	EcoRI-pRIT-R	GAA TTC ACG AAC ACA TAT GGT GCA CTC TCA GTA CAA TCT GCT CTG ATG			
P378	pRIT-EcoRI-P_{comEA}-F	ACC ATA TGT GTT CGT GAA TTC ATG GGA TGC TGT GCA AGG AGA TTT ACC	P_{comEA}-*comEA*	N315 genomic DNA	Create pRIT-P_{comEA}-*comEA-egfp* by Gibson assembly
P379	LinkerA-*comEA*-R	CCG ATC GAT AGC GCT GGT ACC TAT CGT GAA ATA AGA TTT CAG TTT ATC AAA AGT TTT ACT			
P380	pRIT-EcoRI-P_{comFA}1-F	ACC ATA TGT GTT CGT GAA TTC GGT GGA TTA GGT TTA GGC TAT GTT GGC	P_{comFA}1-*comFA*	N315 genomic DNA	Create pRIT-P_{comFA}1-*comFA-egfp* by Gibson assembly
P381	Linker_A-*comFA*-R	CCG ATC GAT AGC GCT GGT ACC TTC ATC AAT CCA ACC TCT TTT TAA TGC TAA TTT GTT CAT			
P398	LinkerA-*mCh*-F	CCG GAA GGA GAT ATA CAT ATG GCT ATC ATT AAA GAG TTC ATG CGC TTC AAA G	*mCh*	pBCB-7-ChK	Create pRIT-P_{comGA}-*comGA-mCh* by Gibson assembly
P399	C-term-R	GCC TCG AGT GGT TGA GCA TGC			
P400	C-term-F	AGG CCT GAT GCA TGC TCA ACC	pRIT-P_{comGA}-*comGA*	pRIT-P_{comGA}-*comGA-egfp*	
P401	*mCh*-LinkerA-R	GCA TGA ACT CTT TAA TGA TAG CCA TAT GTA TAT CTC CTT CCG GCC GAT CGA TAG			
P454	pRIT-EcoRI-P_{comEC}-F	TGC ACC ATA TGT GTT CGT GAA TTC ACA AGG TGT ATC TAC TGA AGG TGC	P_{comEC}-*comEC*	N315 genomic DNA	Create pRIT-P_{comEC}-*comEC-egfp* by Gibson assembly
P455	LinkerA-*comEC*-R	CCG ATC GAT AGC GCT GGT ACC TAA ACC ACT TGC ATT TCC ATA AGA GTT TG			
P456	pRIT-EcoRI-P_{dprA}-F	TGC ACC ATA TGT GTT CGT GAA TTC AAT AAC AGG CGT CTT TAG TAA TGA GGC	P_{dprA}-*dprA*	N315 genomic DNA	Create pRIT- P_{dprA}-*dprA-egfp* by Gibson assembly
P457	LinkerA-*dprA*-R	CCG ATC GAT AGC GCT GGT ACC AAT ATA GTA GTC TTC AAA TAT ATC ATT AGC GTT TAA			
P460	pRIT-EcoRI-P_{ssbC}-F	C ACC ATA TGT GTT CGT GAA TTC ATT CCC ACA TCC CAT TAG TTT AAT ATT TAT GAT TTT TG	P_{ssbC}-*ssbC*	N315 genomic DNA	Create pRIT-P_{ssbC}-*ssbC-egfp* by Gibson assembly

P461	LinkerA-*ssbC*-R	CCG ATC GAT AGC GCT GGT ACC AAT TTC TAA TAA GTC ATG ATT ATC TAT ATT TTG AGA			
P505	*aphA3*-F	GTT TAA GGG CCC ACC TAG GGG TTT CAA AAT CGG CTC CG	*aphA3*	pCNi	Check for kanamycin resistance cassette
P506	*aphA3*-R	CTA TGA CTC GAG GCC GCG GCG CTC GGG ACC CCT ATC TAG C			
P519	*comEA*-pRIT-F	CTG AAA TCT TAT TTC ACG ATA TAA GCA CTA GTG CAG CTT GGC GTA ATC	pRIT-P_{comEA}	pRIT-P_{comEA}-*comEA-egfp*	Create pRIT-P_{comEA}-*egfp-comEA* by Gibson assembly
P520	*egfp*-P_{comEA}-R	GCT AGC CAT TCC TCA ACT TTA TAC ACG TCT GAG CGA GTG			
P521	P_{comEA}-*egfp*-F	CTC AGA CGT GTA TAA AGT TGA GGA ATG GCT AGC AAA GGA GAA GAA CTT TTC ACT GGA	*egfp*	pBCB8-GK	
P522	*comEA-egfp*-R	TAA AAA TTG ATA CAA TAA AAC CAC GCC TCG AGT GGT TGA GCA TGC ATC			
P523	*egfp-comEA*-F	CCA CTC GAG GCG GTG GTT TTA TTG TAT CAA TTT TTA TTA CGC TAT AAA GAT TTT TTA ACT	*comEA*	N315 genomic DNA	
P524	pRIT-*comEA*-R	AAG CTG CAC TAG TGC TTA TAT CGT GAA ATA AGA TTT CAG TTT ATC AAA AGT TTT ACT TCC			
P525	pRIT-P_{comFA}2-F	ATA TGT GTT CGT GAA TTC CAG TTT GAT CAT AAT TCA GTG TTA CTA TAC ATG GTA CTG	P_{comFA}2	N315 genomic DNA	Create pRIT-P_{comFA}2-*comFA-egfp* by Gibson assembly
P526	*comFA*-P_{comFA}2-R	CGA ACT CTC TGT TAT TTT ATA TCT TGT TAC ATT ATC CAT TCG AC			
P527	*comFA*-F	ATG GAT AAT GTA ACA AGA TAT AAA ATA ACA GAG AGT TCG CAA AGT TCA TCA CAA GC	pRIT-*comFA-egfp*	pRIT-P_{comFA}1-*comFA-egfp*	
P528	P_{comFA}2-pRIT-R	TGA ATT ATG ATC AAA CTG GAA TTC ACG AAC ACA TAT GGT GCA CTC TCA GTA CAA TCT G			
P531	SalI-P_{comGA}-F	TGT GTC GAC TGT TCG TGA ATT CGC AGT TGT TGG AGA TAC ATT ATT TAA TAA TGG	P_{comGA}-*comGA-mCh*	pRIT-P_{comGA}-*comGA-mCh*	Create pCNi-P_{comGA}-*comGA-mCh* by ligating SalI-SacI-digested P_{comGA}-*comGA-mCh* insert into SalI-SacI-digested pCNi plasmid
P532	SacI-*mCh*-R	TTA GAG CTC TCA GCC GGC GCG GC			
P533	pCN-X-F	TGG TAT CTT TAT AGT CCT GTC GGG TTT CGC	Variable	All pCN plasmids	Check insertions in pCN plasmids
P534	pCN-X-R	ATT TAG TTT TGG TTC ATC TTC TGT TAA CTT ATT AAC TCT TTC CGC			
P560	*comGC*-FLAG-pRIT-F	GCA AAT GAC TAC AAG GAC GAC GAT GAC AAG TGA GCA CTA GTG CAG CTT GGC	pRIT-P_{comGA}	pRIT-P_{comGA}-*comGA-egfp*	Create pRIT-P_{comGA}-*ComGC*-FLAG by Gibson assembly
P561	*comGC*-P_{comGA}-R	TTG AGT TTT CTT AAG AAA TTT AAA CAT AAA AAC ACC TCC TAC ATA TAA TCA CGT AGG AGG			
P562	P_{comGA}-*comGC*-F	GTA GGA GGT GTT TTT ATG TTT AAA TTT CTT AAG AAA ACT CAA GCG TTT ACA TTG ATA GAG	*ComGC*-FLAG	N315 genomic DNA	
P567	FLAG-*comGC*-R	GTC ATC GTC GTC CTT GTA GTC ATT TGC AAC TGC TTC TCC ATT ACT AAT TGT TAT TGT CTC			

References

1. Monk IR, Tree JJ, Howden BP, Stinear TP, Foster TJ. 2015. Complete Bypass of Restriction Systems for Major Staphylococcus aureus Lineages. MBio 6.
2. Grant SG, Jessee J, Bloom FR, Hanahan D. 1990. Differential plasmid rescue from transgenic mouse DNAs into Escherichia coli methylation-restriction mutants. Proc Natl Acad Sci 87:4645–4649.
3. Morikawa K, Takemura AJ, Inose Y, Tsai M, Nguyen Thi le T, Ohta T, Msadek T. 2012. Expression of a cryptic secondary sigma factor gene unveils natural competence for DNA transformation in Staphylococcus aureus. PLoS Pathog2012/11/08. 8:e1003003.
4. Pereira PM, Veiga H, Jorge AM, Pinho MG. 2010. Fluorescent Reporters for Studies of Cellular Localization of Proteins in Staphylococcus aureus. Appl Environ Microbiol 76:4346–4353.
5. Charpentier E, Anton AI, Barry P, Alfonso B, Fang Y, Novick RP. 2004. Novel Cassette-Based Shuttle Vector System for Gram-Positive Bacteria. Appl Environ Microbiol 70:6076–6085.
