# Supplementary material for: Natural transformation and cell division delay in competent Staphylococcus aureus
Source: Microbiol Spectr. 2023 Oct 13;11(6):e02807-23. doi: 10.1128/spectrum.02807-23 (PMC10714784; doi:10.1128/spectrum.02807-23)
Supplement: Supplemental legends — Legends for Videos S1 to S4. [file spectrum.02807-23-s0002.pdf]

Supplementary videos

Supplementary video 1. 360 ° rotation of a *S. aureus* competent cell with cytoplasmic ComGA-EGFP.

Strain St113 (pRIT-P_{comGA}-*comGA-egfp*) was grown for 25h (10^{-5} dilution) in CS2 medium. ComGA appears in the cytoplasm.

Supplementary video 2. 360 ° rotation of a *S. aureus* competent cell with ComGA-EGFP associated to the inner face of the membrane.

Strain St113 (pRIT-P_{comGA}-*comGA-egfp*) was grown for 22h (10^{-5} dilution) in CS2 medium. ComGA appears associated to the inner face of the membrane.

Supplementary video 3. 360 ° rotation of a *S. aureus* competent cell with a ComGA-EGFP focus.

Strain St113 (pRIT-P_{comGA}-*comGA-egfp*) was grown for 22h (10^{-5} dilution) in CS2 medium. ComGA appears as a focus.

Supplementary video 4. 360 ° rotation of a *S. aureus* competent cell harboring a ComGA-mCherry focus and stained with Vanco-BODIPY.

Strain St228 (pRIT-P_{comGA}-*comGA-mCh*) was grown for 22h (10^{-5} dilution) in CS2 medium and stained with Vancomycin BODIPY FL (Vanco-BODIPY). The cell wall is stained by the Vanco-BODIPY in green and a ComGA-mCherry focus appears in red.
